# Non‐reef habitats in a tropical seascape affect density and biomass of fishes on coral reefs

**DOI:** 10.1002/ece3.6940

**Published:** 2020-11-19

**Authors:** Katie T. Sievers, Eva C. McClure, Rene A. Abesamis, Garry R. Russ

**Affiliations:** ^1^ College of Science and Engineering James Cook University Townsville QLD Australia; ^2^ Australia Research Council Centre of Excellence for Coral Reef Studies James Cook University Townsville QLD Australia; ^3^ Silliman University Angelo King Center for Research and Environmental Management Silliman University Dumaguete City Philippines

**Keywords:** coral reef, fish ecology, marine reserves, ontogeny, seascape, spatial analysis

## Abstract

Nonreef habitats such as mangroves, seagrass, and macroalgal beds are important for foraging, spawning, and as nursery habitat for some coral reef fishes. The spatial configuration of nonreef habitats adjacent to coral reefs can therefore have a substantial influence on the distribution and composition of reef fish. We investigate how different habitats in a tropical seascape in the Philippines influence the presence, density, and biomass of coral reef fishes to understand the relative importance of different habitats across various spatial scales. A detailed seascape map generated from satellite imagery was combined with field surveys of fish and benthic habitat on coral reefs. We then compared the relative importance of local reef (within coral reef) and adjacent habitat (habitats in the surrounding seascape) variables for coral reef fishes. Overall, adjacent habitat variables were as important as local reef variables in explaining reef fish density and biomass, despite being fewer in number in final models. For adult and juvenile wrasses (Labridae), and juveniles of some parrotfish taxa (*Chlorurus)*, adjacent habitat was more important in explaining fish density and biomass. Notably, wrasses were positively influenced by the amount of sand and macroalgae in the adjacent seascape. Adjacent habitat metrics with the highest relative importance were sand (positive), macroalgae (positive), and mangrove habitats (negative), and fish responses to these metrics were consistent across fish groups evaluated. The 500‐m spatial scale was selected most often in models for seascape variables. Local coral reef variables with the greatest importance were percent cover of live coral (positive), sand (negative), and macroalgae (mixed). Incorporating spatial metrics that describe the surrounding seascape will capture more holistic patterns of fish–habitat relationships on reefs. This is important in regions where protection of reef fish habitat is an integral part of fisheries management but where protection of nonreef habitats is often overlooked.

## Graphical abstract

How do multiple habitats in a seascape influence the presence, density, and biomass of coral reef fishes? Although local coral reef habitat is the major influence, non‐reef habitats within 500 m can strongly affect reef fish distributions, especially juvenile fish and wrasses.

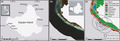

## 1 INTRODUCTION

Fishes use multiple habitats for a variety of ecological reasons. In tropical coral reef ecosystems, nonreef habitats include, but are not limited to mangrove forests, seagrass meadows, and macroalgal beds. Though each habitat offers unique and essential ecosystem services, there is ample and increasing evidence that these nonreef habitats are important to coral reef fishes (Boström et al., 2011; Fulton et al., 2019; Nagelkerken et al., 2015; Pittman & Olds, 2015) and, at least in some places, coral reef fisheries (Fulton et al., 2020; Honda et al., 2013). Diel, tidal, and seasonal migrations of large‐bodied fishes (Haemulids, Lutjanids, and Lethrinids) from coral reefs to seagrass and mangrove habitats to forage and spawn are well documented (Appeldoorn et al., 2009; Honda et al., 2016; Huijbers et al., 2015; Nagelkerken et al., 2000; Verweij et al., 2006). The recruits and juveniles of many reef fish species also use nonreef habitats as nursery grounds to reduce mortality due to predation (Adams et al., 2006; Beck et al., 2001; Dahlgren & Eggleston, 2000; Lefcheck et al., 2019). Juveniles of many coral reef fishes reside in nonreef habitats, often in higher abundances than on coral reefs (Davis et al., 2014; Dorenbosch et al., 2005; Tano et al., 2017). In shallow water tropical seascapes, more than 600 species of coral reef fishes have been found to use adjacent nonreef habitat (Sambrook et al., 2019), yet we still do not understand the full extent of the reliance of coral reef fishes on adjacent nonreef habitats.

The distribution and assemblage structure of fishes on coral reefs can be significantly altered by the spatial configuration of nonreef habitats in the surrounding seascape. Mangroves close in proximity to coral reefs can increase the biomass of reef fishes in the Caribbean (Mumby et al., 2004), and dictate whether some species occur at all on coral reefs (Paillon et al., 2014). Area of adjacent seagrass can have positive relationships with coral reef fish density (Davis et al., 2014; Grober‐Dunsmore et al., 2008). Some studies evaluating spatial connectivity patterns of multiple habitats in a seascape find seascape‐level habitat metrics more influential in describing fish density, diversity, and biomass than within‐patch characteristics of the coral reef (Grober‐Dunsmore et al., 2007; Martin et al., 2015; Mellin et al., 2009; Pittman et al., 2004; Yeager et al., 2011). For example, coral reef fish abundance and distribution in Moreton Bay, Australia, were influenced primarily by proximity to mangroves and seagrass, and only secondarily by local reef characteristics such as coral cover when patches were highly connected (Olds et al., 2012). However, as a relatively new topic in marine systems, results comparing the relative importance of habitat types to fish density at different scales are equivocal, being location‐ and species‐specific. While bottom‐up effects of coral reef benthic habitat are an essential driver in coral reef fish distributions (Russ et al., 2015), including surrounding habitat, metrics is a necessary and productive avenue to improve our understanding of species–habitat interactions across diverse seascapes.

To counteract the uncertainty in species–habitat use patterns, adopting a hierarchical, multi‐scale approach enables evaluation of species–habitat relationships at both the local (within patch) and seascape (across patches) scales (Berkström et al., 2012; Mellin et al., 2009; Pittman & Brown, 2011; Wedding et al., 2011). Remote sensing technology and spatial analysis software have allowed for the development of marine habitat maps that describe diverse seascapes in high resolution across large spatial extents (Hedley et al., 2016; Kendall & Miller, 2008; Roelfsema et al., 2018). This provides users with the flexibility to explore species–habitat relationships across multiple spatial scales, at spatial resolutions that are useful for ecological studies.

In the Philippines, coral reefs are often adjacent to or near large areas of seagrass beds, macroalgal beds, and/or mangrove stands. We use this system to explore how spatial connectivity of multiple habitats in a seascape affects coral reef fish. The Philippines is the northern tip of the Coral Triangle, and is considered a global biodiversity and conservation hotspot for shallow water reef fishes (Carpenter & Springer, 2005; Nañola et al., 2011), with the highest concentration of no‐take marine reserves (NTMR) in the world (Cabral et al., 2014; Horigue et al., 2012). However, these NTMRs are mostly placed on coral reefs, often neglecting adjacent habitats (Weeks et al., 2010). We aim to understand fish–habitat relationships in a diverse model seascape, specifically focusing on coral reef fishes to explore 1. the relative importance of local scale coral reef habitat and adjacent nonreef habitats on fish species presence, density, and biomass, and 2. which nonreef habitats and spatial connectivity metrics are the most important.

## 2 METHODS

### 2.1 Study location

**Figure 1:**
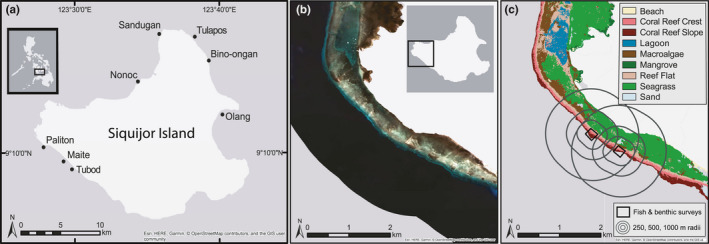
Survey sites and mapping process of submerged habitat on Siquijor Island. (a) Map of sites (black circles) where fish and benthic assemblages were surveyed on coral reefs in April–July 2016. (b) Satellite imagery from one area of western Siquijor Island (San Juan) from the Planet imagery at 3‐m resolution showing the true color image. (c) Map of classified habitats derived from satellite imagery including survey locations of coral reef substrate and fish, and radii scales (250, 500, 1,000‐m) from which spatial metrics of adjacent habitat types were calculated

This study was conducted around Siquijor Island in the Visayan region of the Philippines (Figure 1a). Shallow water benthic habitats of Siquijor include macroalgal beds, mangroves, and seagrass beds of varying spatial extent adjacent to fringing coral reefs. Seagrass meadows in Siquijor are composed of a diverse grouping of *Cymodocea rotundata, C. serrulata*)*, Halodule pinifolia, H. uninervis, Thalassodendron ciliatum, Enhalus acroides, Halophila beccarii, H. minor, H. ovalis, H. spinulosa,* and *Thalassia hemprichii* (Meñez et al., 1983). Macroalgal beds are characterized by *Sargassum* spp. when it is dominant and smaller red and green understory macroalgae when the *Sargassum* canopy has senesced seasonally. Mangrove habitats are patchily distributed around the island, composed mainly of *Rhizophora* spp. that were replanted between the mid‐1980s and early 1990s (De Leon & White, 1999), with some remaining natural stand of *Sonneratia* spp. and *Avicennia* spp. As of 2018 Siquijor had 12 NTMRs, providing an ideal location to evaluate the effects of nonreef habitat and NTMRs on coral reef fish presence, density, and biomass.

### 2.2 Fish and habitat surveys

Surveys of reef fish and benthos were conducted in April–July 2016 at eight locations around Siquijor Island (Figure 1a), with paired NTMR and control (open to fishing) sites, totaling to 16 sites. Location selection was based on distance to nonreef habitat, accessibility, coral reef habitat type, and NTMR compliance. Underwater visual censuses (UVC) were conducted to quantify the fish and benthic communities on coral reefs. At each location, three or four transects were surveyed along both the coral reef slope and reef crest per site, representing 6 or 8 transects per location, and totaling to 108 transects across all locations. The number of replicate transect surveys was determined by the NTMR size. Along a 50 m by 5‐m transect, large mobile reef fish (>10 cm TL) were counted and sized to the nearest centimeter. On the return swim, smaller (≤10 cm TL) reef fish species were recorded within a 2‐m width. Biomass of fishes was calculated using published length–weight relationships (Kulbicki et al., 2005). For benthic surveys, substratum was identified at 50‐cm intervals along the 50‐m transect and was classified based on substrate (rock, sand, rubble, coarse sand) and benthic cover (abiotic, crustose coralline algae, epilithic algal matrix, macroalgae, soft coral, hard coral, other) (Table 1). Macroalgae and soft coral were identified to genus when possible. Hard coral was identified to genus and classified into growth form (fragile, robust). The “other” category included sessile invertebrates such as sponges, tunicates, and gorgonians. Structural complexity was estimated visually on a 0–5 scale following methods used in Wilson et al. (2007). In general terms, 0 = flat and 5 = highly complex structure.

**Table 1:** Predictor variables used for model analysis with their mean, minimum, and maximum values from coral reef surveys and spatial analysis output

Variable	Unit	Mean	Minimum	Maximum
*Local Reef Category*				
Rubble	% Cover	24	0	91
Sand	% Cover	16	0	83
Macroalgae	% Cover	10	0	38
Epilithic algal matrix (EAM)	% Cover	32	5	87
Soft coral	% Cover	8	0	38
Hard coral	% Cover	26	1	81
Fragile coral	% Cover	11	0	61
Robust coral	% Cover	15	1	47
Depth	Meters	9.6	2.9	17.5
Structural complexity	Scale 0–5	2.7	0	5
*Adjacent Habitat Category*				
Distance to shore	Meters	209	63	477
Distance to seagrass	Meters	88	5	650
Distance to macroalgae	Meters	35	5	100
Distance to mangrove	Meters	2,380	104	8,200
Coral reef area within 500 m*	% Area	26	13	41
Macroalgal area within 500 m*	% Area	14	5	41
Mangrove area within 500 m*	% Area	2	0	10
Reef flat area within 500 m*	% Area	11	1	24
Seagrass area within 500 m*	% Area	32	0	58
Sand area within 500 m*	% Area	12	1	20

Note

Variables are separated by scale category (local reef or adjacent habitat). * denotes radii measures were only reported for the 500‐m radius, but were also calculated for 250 and 1,000‐m spatial radii.

### 2.3 Habitat mapping

Remotely sensed satellite imagery paired with in situ georeferenced habitat data were used to create a marine benthic habitat map. Images from the GeoEye and PlanetScope satellite sensors were acquired from the Digital Globe Foundation, and Planet, respectively. The GeoEye satellite provides a spatial resolution of 1.84‐m, and Planet provides a 3‐m resolution, both across four spectral bands of blue, green, red, and near‐infrared (NIR) (Figure 1b). Both sensors were necessary to acquire complete coverage of the island. Preprocessing of imagery was conducted using the software ENVI (v. 5.3, Harris Geospatial Inc.). Band ratios were calculated to provide additional unique spectral signatures for benthic habitat classes (Phinn et al., 2012; Roelfsema et al., 2013). Band ratios were the following: blue to red (B/R), blue to green (B/G), and red to NIR (R/NIR). After preprocessing, classification of imagery into habitat types was conducted using the maximum‐likelihood classification tool in ArcGIS, v. 10.4.1. Feature classes were a combination of biotic and geomorphological features: seagrass meadows, macroalgal beds, reef flat, reef crest, reef slope, lagoon, sand, mangrove forest, and beach (Figure 1c). Georeferenced habitat data points (*n* = 500) collected in situ in 2016–2018 informed the maximum‐likelihood classification, with 70% of points used for training, and the remaining 30% used for validation of the classified map. The map was then manually reviewed and edited for obvious errors, smoothed using the majority filter in ArcGIS, and converted to polygons for spatial analysis. Map validation identified 72% accuracy of habitat classification using the maximum‐likelihood method.

### 2.4 Spatial analysis

Fish and benthic survey locations were overlaid onto the classified habitat map to calculate spatial statistics of the seascape surrounding each site (*n* = 16). Adjacent habitats used for spatial analysis were seagrass, macroalgae, sand, reef flat, and mangroves. For each location, distance to the nearest habitat type was measured using edge‐to‐edge distance between survey sites and each habitat. Because reef fish species respond to benthic habitat at varying spatial scales, we used a multi‐scale approach to measure the area of each habitat (Grober‐Dunsmore et al., 2009). Buffer zones surrounding each survey site were calculated at three different spatial scales (250, 500, 1,000‐m) (Figure 1c). Buffers were clipped by shore and deepwater features to only represent shallow water habitat. The proportion of each habitat within each buffer zone was calculated as the area of habitat divided by the total area of the clipped buffer. These data were then incorporated with the benthic survey data on coral reefs for further analysis (Table 1). Global Moran's I was calculated for the 500‐m habitat spatial scale to evaluate any potential spatial autocorrelation. Spatial data were not significantly spatially autocorrelated for the 500‐m scale (Moran's I = 0.370, *p* = .24).

### 2.5 Statistical analysis

Boosted regression trees (BRT; Elith et al., 2008) were used to evaluate how benthic habitats at different spatial scales affected coral reef fishes using the gradient BRT method from the gbm package. BRTs are an excellent tool to understand the relative influence of multiple predictor variables, with the advantage of handling multi‐collinearity and nonlinearity among predictor variables (De’ath, 2007). Fish groups were analyzed in terms of density and biomass, or presence/absence, using Poisson, Gaussian, and Bernoulli distributions, respectively. Presence/absence was used for species groups with too few observations for density and biomass analysis (Lutjanidae and Serranidae). In total, 32 BRT models were run on fish groups with the greatest number of observations at the family level: Labridae (wrasses, excluding parrotfishes), Lutjanidae (snappers), Serranidae (groupers), Pomacentridae (damselfishes), Chaetodontidae (butterflyfishes), and Acanthuridae (surgeonfishes) (Table 2). Parrotfishes (Labridae, subfamily Scarinae) were run at the level of genus for two different feeding‐type groups, *Scarus* and *Chlorurus*, where Scarus are scrapers and Chlorurus excavators. *Hipposcarus* was included in the "Scarus" group, and *Cetoscarus* was included in the "Chlorurus" group based on their feeding modes. Models for juvenile reef fish density were only possible for wrasses, and the parrotfish groups *Scarus* and *Chlorurus,* due to the lack of juveniles, observed from other families. Fish groups were also separated by coral reef zones, that is, reef crest and slope.

**Table 2:** Summary of each reef fish group with model parameters selected for bootstrap boosted regression tree analysis using the gbm step method

Model	Species Group	Stage	Metric	Level	Ave Trees	95% CI Trees	Ave CV Deviance	95% CI CV Deviance
1	Surgeonfishes	Adult	Biomass	Crest	4,406	573, 10,000	0.51	0.15, 0.87
2	Surgeonfishes	Adult	Biomass	Slope	7,123	800, 10,000	0.27	0.07, 0.74
3	Surgeonfishes	Adult	Density	Crest	4,457	873, 10,000	0.71	0.45, 0.87
4	Surgeonfishes	Adult	Density	Slope	8,444	992, 10,000	0.50	0.07, 0.89
5	Butterflyfishes	Adult	Biomass	Crest	3,376	450, 10,000	0.61	0.35, 0.89
6	Butterflyfishes	Adult	Biomass	Slope	6,317	600, 10,000	0.34	0.07, 0.75
7	Butterflyfishes	Adult	Density	Crest	6,904	2,595, 10,000	0.63	0.45, 0.73
8	Butterflyfishes	Adult	Density	Slope	3,444	400, 9,702	0.60	0.29, 0.85
9	*Scarus*	Adult	Biomass	Crest	7,947	1,245, 10,000	0.31	0.06, 0.59
10	*Scarus*	Adult	Biomass	Slope	6,716	892, 10,000	0.31	0.02, 0.57
11	*Scarus*	Adult	Density	Crest	6,861	1,390, 10,000	0.29	0.02, 0.58
12	*Scarus*	Adult	Density	Slope	8,614	1,100, 10,000	0.33	0.02, 0.59
13	*Scarus*	Juvenile	Density	Crest	2,972	523, 9,760	0.69	0.45, 0.84
14	*Scarus*	Juvenile	Density	Slope	2,265	400, 9,155	0.52	0.23, 0.74
15	*Chlorurus*	Adult	Biomass	Crest	3,134	300, 10,000	0.41	0.07, 0.74
16	*Chlorurus*	Adult	Biomass	Slope	4,145	397, 10,000	0.46	0.15, 0.78
17	*Chlorurus*	Adult	Density	Crest	2,720	300, 9,160	0.35	0.1, 0.64
18	*Chlorurus*	Adult	Density	Slope	3,789	621, 10,000	0.48	0.06, 0.82
19	*Chlorurus*	Juvenile	Density	Crest	3,691	261, 10,000	0.31	0.02, 0.65
20	*Chlorurus*	Juvenile	Density	Slope	1,404	205, 9,895	0.24	0.01, 0.6
21	Wrasses	Adult	Biomass	Crest	7,712	4,206, 10,000	0.71	0.42, 0.91
22	Wrasses	Adult	Biomass	Slope	5,296	370, 10,000	0.64	0.35, 0.89
23	Wrasses	Adult	Density	Crest	7,577	2,407, 10,000	0.28	0.06, 0.52
24	Wrasses	Adult	Density	Slope	4,792	600, 10,000	0.51	0.09, 0.78
25	Wrasses	Juvenile	Density	Crest	4,264	1,182, 9,730	0.62	0.35, 0.85
26	Wrasses	Juvenile	Density	Slope	4,955	1,250, 10,000	0.49	0.16, 0.79
27	Damselfishes	Adult	Density	Crest	3,306	300, 9,976	0.55	0.17, 0.86
28	Damselfishes	Adult	Density	Slope	2,847	423, 6,126	0.74	0.36, 0.95
29	Snappers	Adult	Presence	Crest	4,431	1,340, 10,000	0.29	0.07, 0.56
30	Snappers	Adult	Presence	Slope	1744	650, 3,347	0.44	0.22, 0.72
31	Groupers	Adult	Presence	Crest	1719	450, 4,076	0.50	0.26, 0.8
32	Groupers	Adult	Presence	Slope	1,373	371, 4,008	0.40	0.18, 0.71

Note

Mean trees and Mean CV deviance are reported values from the bootstrap (sample and replacement) process with their upper and lower 95% confidence limits.

To identify the scale at which reef fish responded to the seascape, a BRT was run for each adjacent habitat type at all three spatial scales (250, 500, 1,000 m) for each response variable. The "best" scale for each habitat type was selected as the radius with the highest relative importance, and only that scale was included for further analysis. Variables with correlation values >0.8 (e.g., hard coral, fragile coral, robust coral) were run in a BRT, and only the variable with highest relative importance was selected for the remaining analysis. Full models were then run with these preselected variables with an interaction depth of 3 and bag fraction of 0.75 using the gbm.step method in the gbm package, and were calibrated for best results by altering the learning rate to achieve the optimal number of iterations between 1,000–10,000 trees, based on a 10‐fold cross‐validation procedure. The gbm.simplify process was used to reduce the number of variables by an iterative backwards stepwise removal of the least influential variables using k‐fold cross‐validation until the change in predictive deviance was minimized. The simplify process selected the nine most influential variables, and NTMR status was the tenth variable to evaluate any reserve effect. To account for stochasticity and incorporate uncertainty values for relative importance, models were bootstrapped (sampling with replacement) 100 times. Error in relative importance and deviance explained values were measured by 95% confidence intervals from the bootstrapping process. Cross‐validation deviance (CV deviance) was calculated by subtracting the CV deviance from the null deviance and dividing by the null deviance. Mean relative importance was used as an indicator for variable importance. Because models had 10 variables, relative importance values greater than 10% were considered influential as they were selected more frequently than expected by chance. The mean relative importance was summarized only for influential variables (>10% relative importance) and compared between variables categories (local reef vs. adjacent habitat) (Table 1). Here, we define “local reef” as the small‐scale benthic habitat characteristics of a coral reef, whereas “adjacent habitat” describes larger scale spatial metrics of multiple habitat types across a seascape. Wilcoxon ranked tests for nonparametric data were used to compare the mean relative importance between local reef and adjacent habitat categories across all models, at the level of reef zones (crest and slope), fish life stages (juvenile and adult), and for each fish group.

## 3 RESULTS

**Figure 2:**
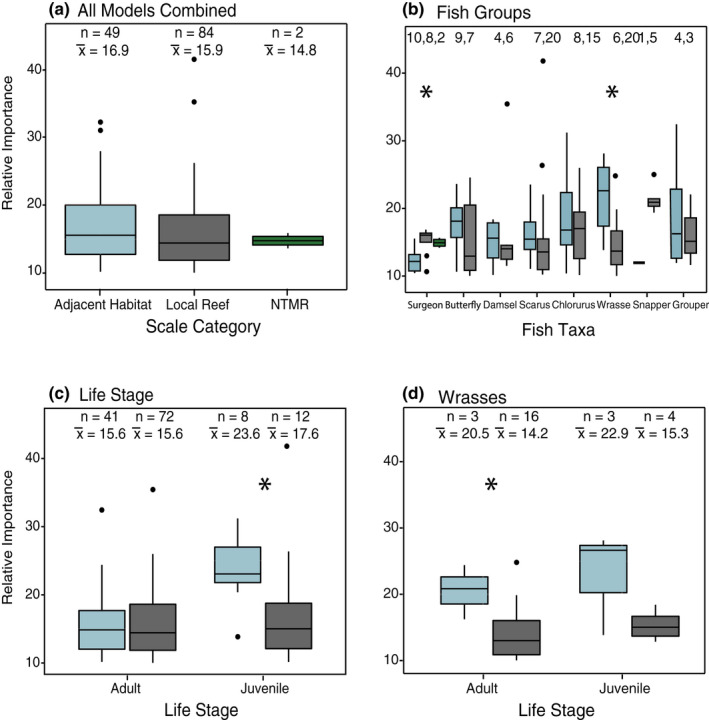
Relative importance of variables with high influence (>10% relative importance) comparing the difference between scale categories of adjacent habitat (blue) and local reef habitat (gray), and no‐take marine reserve (NTMR) effect (green) for models describing density, biomass, and presence of coral reef fish. Boxplots show medians and quartiles; dots are outliers. * indicates significance of relative influence between scale categories based on Wilcoxon rank‐sum tests. Values at the top of each plot show the number of times each variable was included in the model (*n*) and the mean relative importance value ($\bar{x}$) of the variable categories across models for (a.) all models combined, (b) separated by fish taxa, (c) life stage, and (d) for juvenile and adult wrasses

For all 32 BRT models explaining reef fish presence, density, or biomass, 62.2% of the influential variables were local coral reef variables, 36.3% were adjacent habitat metrics, and 1.5% were NTMR variables. The mean relative importance of influential variables (>10% relative importance) between local reef and adjacent habitat was similar (16.9 and 15.9, respectively; Figure 2a) and not statistically different (Wilcoxon rank‐sum test, W = 2,306, *p* = .248) (Table 3). For surgeonfish, local reef variables had significantly higher mean relative importance in determining density and biomass compared to adjacent habitat (Wilcoxon rank‐sum test, W = 11, *p* = .011) (Figure 2b). In contrast, the mean relative importance of adjacent habitat was significantly higher for wrasses (Wilcoxon rank‐sum test, W = 104, *p* = .008). The remaining taxa had no significant differences in mean relative importance between the two habitat categories. Juvenile fish density (represented by *Chlorurus, Scarus*, and wrasses combined) had significantly higher mean relative importance for adjacent habitat variables (Wilcoxon rank‐sum test, W = 78, *p* = .022) (Figure 2c). For wrasses, both juvenile and adult density had greater mean relative importance for adjacent habitat variables (Figure 2d).

**Table 3:** Wilcoxon rank‐sum tests for variables >10% relative influence comparing the values between habitat categories (local reef vs. adjacent habitat) for each grouping

Comparison grouping	*W*	*p* value
Overall	2,306	.248
Crest	531	.232
Slope	613	.743
Adult	1,487	.950
**Juvenile**	**78**	.022*
Biomass	240	.711
Density	793	.144
Presence	14	.421
**Surgeonfishes**	**11**	.011*
Butterflyfishes	40	.397
*Chlorurus*	68	.628
**Wrasses**	**104**	.008*
Wrasses–juvenile	9	.378
**Wrasses–adult**	**42**	.050*
Snappers	0	.242
Damselfishes	12	1.000
*Scarus*	96	.158
Groupers	7	.860

Note

Bold and * indicate significant difference.

**Figure 3:**
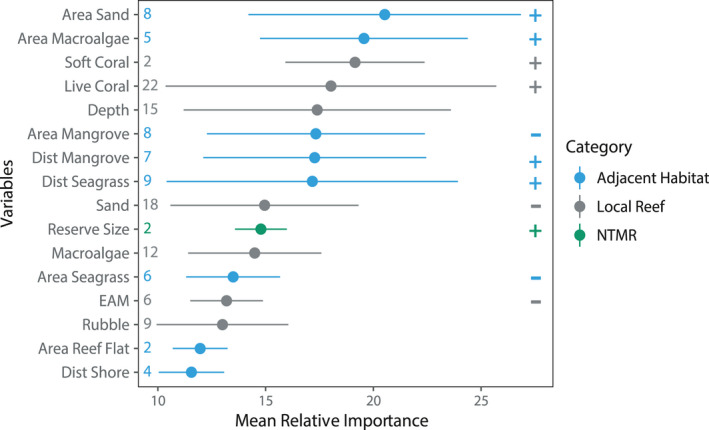
Mean relative importance of variables with high influence (>10% relative importance) across all 32 reef fish models analyzed for density, biomass, and presence of coral reef fish. Dots represent means, and bars represent upper and lower standard deviation. Colors indicate the scale category for adjacent habitat (blue), local reef (gray), or no‐take marine reserve (green). Values on the left‐hand side of the graph represent the number of times that variable was used in a model, and symbols (+ or −) on the right‐hand side indicate the direction of the relationship when obvious

Individual variables with the highest mean relative importance were adjacent habitat metrics of sand and macroalgae (Figure 3). Both adjacent sand and macroalgae had a consistently positive relationship with fish taxa responses, where the greatest change occurred between 10% and 20% coverage in the surrounding seascape. The most selected radius for adjacent habitat variables was the 500‐m spatial scale for all habitats except seagrass, which was dominated by the 1000‐m spatial scale (Table 4). Local coral reef variables were found to strongly affect reef fish presence, density, and biomass, and were included 1.7 times more frequently than adjacent habitat variables. Specifically, live coral cover (selected in 69% of models, Figure 3) was a consistent, strong, and positive predictor of coral reef fish presence, density, and biomass for most models. Percent cover of sand (56%) and depth (47%) were also influential local reef variables with sand having a negative effect and depth having mixed effects.

**Table 4:** Summary of radii selection for adjacent habitat variables

Radius	Macroalgae	Mangrove	Seagrass	Reef Flat	Sand	Total
250	0	0	0	1	1	2
500	4	8	0	1	6	19
1,000	1	0	6	0	1	8
Total	5	8	6	2	8	29

Note

Values reported are only for influential variables with a relative importance > 10% in final BRT models.

For juvenile fish, adjacent habitat variables had higher relative importance compared to local coral reef variables (Figure 2c). Wrasse and *Chlorurus* juveniles were most influenced by adjacent sand in the seascape (positive relationship), and adjacent macroalgal habitat (positive) (Appendix S1). *Scarus* juveniles were strongly positively influenced by percent fragile coral but were secondarily influenced by the adjacent habitat variables distance to mangrove (positive relationship) and amount of sand within 500 m (positive relationship). Across all juvenile BRT models, percent cover of sand at the local reef scale was the most frequently selected variable (5 of 6 models) with a negative relationship, followed by a positive relationship with percent cover of fragile coral (4 of 6 models).

**Figure 4:**
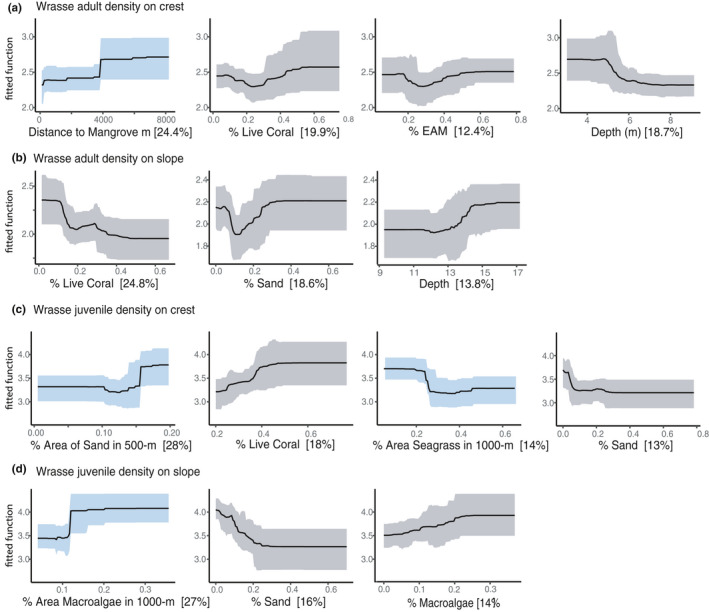
Partial dependence plots from boosted regression tree (BRT) bootstrap analysis for wrasse adult density on crest (a) and slope (b), and wrasse juvenile density models on the crest (c) and slope (d) and with the relative importance of each variable in brackets [%]. Partial plots present the relationship of each variable when all other variables are at their mean. Center line is the mean, and ribbons are 95% confidence intervals for 100 bootstrap runs. Blue ribbons are for adjacent habitat variables, and gray ribbons are for local reef variables. For each model, only partial plots with high relative importance (>10%) are shown

Wrasses were the only fish group to have higher relative importance of adjacent habitat spatial metrics for both adults and juveniles (Figure 2d). For wrasses, the most influential variables were adjacent macroalgae, adjacent sand, and distance to mangrove, all with positive relationships to wrasse density and biomass (Figure 4) (Supplementary material Appendix S1, models 21–26). However, local reef variables did have the greatest inclusion rate in wrasse models, where the percent cover of sand (negative relationship) and percent live coral cover (mixed relationships) were selected most frequently. *Scarus* juvenile density was positively affected by fragile coral cover at the local reef scale, but adult density and biomass was predominantly influenced by macroalgal cover at the local reef scale (mixed effects), and negatively influenced by distance to mangrove and seagrass (Appendix S1, models 9–14). In *Chlorurus* models (models 15–19), live coral cover on the local reef positively influenced adults, whereas juveniles were positively influenced by the amount of adjacent sand in the surrounding seascape. The area of seagrass in the surrounding seascape was also a common predictor in *Chlorurus* models, appearing in three of six models with a negative response to area of adjacent seagrass. For snapper presence (models 31–32), influential variables were almost all local reef. For surgeonfish (models 1–4), NTMR size was included as an influential predictor, positively affecting density and biomass of fish on the reef crest and was the only fish group to have an NTMR variable selected as influential. For damselfish density (models 27–28), the reef crest model was influenced by depth, whereas the reef slope model was influenced by distance to seagrass and mangrove. For grouper presence (models 31–32) on the reef crest there was high importance of adjacent habitat variables, whereas on the reef slope, the presence of groupers was influenced by local coral reef variables. Finally, for butterflyfishes (models 5–8), mangrove variables were present in all models, with a negative influence on density and biomass of fish.

## 4 DISCUSSION

Overall, reef fish presence, density, and biomass were affected primarily by local within‐reef attributes and were secondarily influenced by adjacent habitat in the seascape. Although local reef variables were selected most often in models, the mean relative importance for influential variables was similar between adjacent habitat and local reef. This implies that reef fishes in our seascape are responding to features beyond their immediate vicinity and that adjacent habitat measures of the seascape at the scale of hundreds of meters are important to consider. Other research comparing the influence of local coral reef and adjacent habitats on coral reef fishes has found that seascape‐scale habitat can be more important than local reef habitat (Henderson et al., 2017; Kendall et al., 2011; Knudby et al., 2011; Olds, et al., 2012; Yeager et al., 2011). In high connectivity seascapes, fish assemblages can be more similar between coral reefs and nonreef habitats compared to more isolated seascapes (van Lier et al., 2018), and many species traditionally classified as coral reef dwellers are also found in other nonreef habitats (Evans et al., 2014; Sambrook et al., 2019). Here, we find that on coral reefs, juvenile *Chlorurus* and wrasse were more strongly influenced by adjacent habitat metrics than local reef factors. For adjacent habitat, the relative amount of sand and macroalgae in the surrounding seascape were the strongest predictors of reef fish density and biomass, with seagrass and mangrove habitat having a lesser effect.

One of the most influential habitats in our models was sand, where sand adjacent to coral reefs had a positive effect on fish, while sand at the local reef scale had a negative effect (e.g., Figure 4). Adjacent sand in the seascape had the highest average relative importance of any habitat metric, with a consistent, positive change in density and biomass of reef fish when sand was between 10%–20% of the total area of a seascape. In Siquijor, sand in the seascape was in the back reef areas, on the reef slope, and as large sand patches interspersed throughout the seascape. We hypothesize that a low amount of sand cover between 10%–20% may represent the presence of transition zones to other important habitats. These transition zones, or ecotones, have their own unique contribution that mediates species distributions and interactions, and can be an important seascape predictor (Pittman et al., 2007; Valentine et al., 2007; Vanderklift et al., 2007). An alternative hypothesis would be an isolation effect, where sand patches adjacent to coral reefs reduce the overall area of preferred habitat, thus concentrating fish on coral reefs. At small spatial scales, isolated reefs can have increased densities of fishes (Belmaker et al., 2005; Chittaro, 2002), and sandy habitat adjacent to coral reefs can alter movement of fishes (Turgeon et al., 2010). Contrastingly, sand at the local reef scale had a negative relationship with fish density and biomass. Although some taxa may benefit from the presence of sand and rubble at a local scale (e.g., parrotfishes and wrasses) (Russ et al., 2015, 2017), other taxa which are more reliant on the reef structure itself may respond negatively to sand and rubble (e.g., damselfishes and butterflyfishes) (Russ & Leahy, 2017). However, responses to sand on transects can be taxon‐specific, where species responses vary even within the same family (Lowe et al., 2019; Russ et al., 2017, 2018). Here, the opposing relationship of sand cover at different spatial scales underpins the importance of employing a multi‐scale approach to describing fish–habitat relationships.

Interestingly, we found that fishes were negatively associated with mangrove and seagrass habitats, where density and biomass of fishes on coral reefs were highest when these habitats were farther away and made up less of the seascape. This is counter to other seascape studies which show increased coral reef fish presence and biomass with increased spatial connectivity to seagrass and mangrove habitat (Mumby et al., 2004; Nagelkerken et al., 2002; Olds et al., 2013; Verweij et al., 2006). In this Philippine system, the tidal regime makes these habitats inaccessible for significant periods of time, which may limit their use by coral reef fishes. Indeed, the importance of mangroves has been shown to be strongly tidally influenced (Lee et al., 2014), and mangroves play a larger role for juvenile fishes in regions where mangrove stands are permanently inundated (Igulu et al., 2014). Philippine mangrove systems have been considerably altered, by cutting, coastal development, and planting (Primavera & Esteban, 2008). While planting can increase the extent of mangrove stands, it may come at a cost to their ecological function if mangrove species are planted in unsuitable habitats (e.g., *Rhizophora* spp. planted on seagrass beds) (Lee et al., 2014; Primavera & Esteban, 2008). Potentially, planted mangrove habitats in our study seascape may not sufficiently mimic natural ecological systems, partially accounting for the negligible effects of adjacent mangroves on fish dynamics on coral reefs.

For juvenile wrasses and juveniles in the parrotfish genus *Chlorurus*, adjacent nonreef habitat metrics had a significantly higher mean relative importance. We believe that the present study is one of the first examples to show that the surrounding seascape influences juveniles of some fish genera on coral reefs. Nonreef habitats are sometimes important nursery grounds for coral reef fish (Adams et al., 2006; Cocheret De La Morinière et al., 2002; Sheaves et al., 2015). Higher densities of juvenile reef fishes in nonreef habitats compared to coral reefs implies their nursery value to coral reef fish populations (Kimirei et al., 2015; Nagelkerken et al., 2000; Tano et al., 2017). Nonreef habitats are suggested to be optimal nursery habitat for juveniles due to reduced predation risk (Dahlgren & Eggleston, 2000; Dorenbosch et al., 2009; Valentine et al., 2007), and often greater availability of food resources (Kramer et al., 2015; Tano et al., 2016). Coral reefs with high spatial connectivity to adjacent habitats may be benefitting from ontogenetic habitat shifts of fishes from adjacent nonreef nursery habitat. Exploring the relative abundance of juveniles on both coral reef and nonreef habitats would further validate these hypotheses and should be explored in more detail.

Juvenile wrasse and juvenile *Chlororus* densities on coral reefs had a positive relationship with macroalgae and sand in the surrounding seascape. In our study system, macroalgal beds occur around the entire island, and we suggest that for Siquijor, macroalgal beds may be critical juvenile nursery habitat for some coral reef fish species. Recent evidence suggests that *Sargassum* dominated macroalgal beds harbor significantly greater densities of juvenile fishes compared to other nonreef habitat such as seagrass (Eggertsen et al., 2017; Fulton et al., 2019; Tano et al., 2017). However, for fishes, the ecological importance of sand in the seascape is less clear. Some parrotfish species are known to occasionally forage in sandy areas (Russ, 1984) and have been shown to preferentially associate with sand and soft unvegetated habitat during the juvenile stage (Mellin et al., 2007). Comparatively, *Scarus* juveniles were strongly influenced by the amount of live fragile coral cover on reefs (e.g., branching *Acropora* and *Porites*) rather than by adjacent habitat. Juvenile *Scarus* parrotfish have been one of the more conspicuous taxa observed in nonreef habitats (Gullström et al., 2011; Sambrook et al., 2019; Tano et al., 2017), but have also been shown to associate with small branching pocilloporid corals (Bellwood & Choat, 1989) and dead coral skeletons in back reef habitats (Wilson et a l., 2010). Perhaps the discrepancy between species within the parrotfish family (*Chlorurus* and *Scarus*) is demonstrating multiple postsettlement habitat selection strategies and/or multiple ontogenetic habitat shifts. Though research has detailed how ontogenetic shifts occur by changes in diet (Bellwood, 1988; Chen, 2002), home range (Streit & Bellwood, 2017; Welsh et al., 2013), and habitat use (Dahlgren & Eggleston, 2000), further research must explore how habitat use patterns and ontogenetic shifts may be modified by the habitat availability and spatial configuration of the seascape.

Wrasses were the only fish group to show a significantly greater relative importance of adjacent habitat variables than local reef variables for both juveniles and adults. The amount of adjacent macroalgae and sand in the seascape were both positively correlated with wrasse density and biomass on coral reefs (Figure 4). Wrasses have been highlighted as a group with a high prevalence for multi‐habitat use (Sambrook et al., 2019) and can respond to seascape‐level spatial dynamics (Staveley et al., 2017). Van Lier et al. (2018) showed greater overlap in wrasse assemblage structure between coral reefs and macroalgal beds when macroalgal beds were close to coral reefs with *Thalassoma* generalists identified as driving this response. Generalist species are more versatile in their diet and ability to use different habitats, and generalist wrasse species are more likely to move across a wider range of benthic resources compared to their specialist counterparts (Berkström, et al., 2012; Berkström et al., 2014). This plasticity may allow individuals to take advantage of nearby nonreef habitats such as macroalgal beds, which can have higher abundances of epifauna, small crustaceans, and copepods, potential dietary sources for many tropical wrasses (Berkström, et al., 2012; Kramer et al., 2015; Tano et al., 2016). Nonetheless, live coral cover was also an important factor affecting wrasses, selected as an influential predictor in 5 out of 6 BRT models. Thus, our results indicate that both local reef variables such as live coral cover, as well as adjacent habitat variables influence density of wrasses. Indeed, wrasses in the Philippines have been shown to correlate with benthic dynamics, mirroring long‐term changes in benthic substrata (Russ et al., 2017). However, those responses were taxon‐specific, varied, and occurred on small offshore Philippine islands with little to no shallow adjacent nonreef habitats.

In a complex island seascape in the Philippines, density, biomass, and presence of coral reef fishes were driven by both local reef habitat on coral reefs, and adjacent habitats in the surrounding seascape. Adjacent habitats were the primary driver for some fish taxa (e.g., wrasses) including their juveniles. We found that coral reef fishes responded to adjacent habitats across multiple spatial scales but measuring the surrounding seascape at a 500‐m scale obtained the best model results. Employing a multi‐scale approach better explained reef fish patterns and incorporation of multiple adjacent habitats across an island seascape may offer deeper insights into the structuring of coral reef fish assemblages. This is especially relevant for regions like the Philippines where nonreef habitats are heavily impacted by coastal development, fishing pressure, and pollution, and where juvenile fishes are often the direct or incidental targets of fisheries. When considering management strategies that are spatially focused, such as NTMRs, adopting a multi‐scale seascape‐level approach would consider other nonreef habitats that can often be overlooked in the management process (Weeks et al., 2010).

Interestingly, surgeonfishes were the only species group to include influential NTMR effects in models. Results of surgeonfish responses to NTMRs in the Philippines have been varied (Russ et al., 2018). Yet, very few studies have focused on the interaction between seascapes and NTMR effects (Olds et al., 2016) and this topic should be pursued further.

Developing NTMR networks to improve reserve performance and region‐wide resilience has been a major focus in recent years (Gaines et al., 2010; Weeks et al., 2014). Incorporating nonreef habitats in the establishment of NTMR networks could better conserve populations for species of reef fish with ontogenetic migrations (Green et al., 2015; Grüss et al., 2011). Accounting for ecological processes such as ontogenetic habitat shifts and movement patterns could greatly increase the conservation potential of NTMRs to improve fish species diversity, abundance, and biomass (Brown et al., 2016; Engelhard et al., 2017, Olds et al. 2016). For fishes that utilize nonreef habitats, adjacent habitats can even outweigh the NTMR effect for adult fish biomass on coral reefs (Nagelkerken et al., 2012), or act synergistically with NTMRs to improve NTMR outcomes (Olds et al., 2012). Indeed, nonreef habitats in the Philippines were identified as priority conservation areas to “optimize tradeoffs between biodiversity and fishery targets” (Weeks et al., 2010). With the improved ability to obtain satellite imagery and map habitats, incorporating simple metrics such as distance to adjacent habitats and total area of multiple habitats is now much more attainable. We argue that including habitat metrics across multiple spatial scales to describe reef fish patterns, dynamics, and functions should be considered when feasible, and is especially critical in diverse seascapes.

## CONFLICT OF INTEREST

There are no competing interests with any authors.

## AUTHOR CONTRIBUTION

**Katie T Sievers:** Conceptualization (lead); Data curation (equal); Formal analysis (lead); Funding acquisition (equal); Methodology (lead); Writing‐original draft (lead); Writing‐review & editing (equal). **Eva C. McClure:** Conceptualization (supporting); Data curation (equal); Funding acquisition (equal); Methodology (supporting); Writing‐review & editing (equal). **Rene Aberin Abesamis:** Project administration (equal); Supervision (supporting); Writing‐review & editing (equal). **Garry R Russ:** Funding acquisition (equal); Project administration (equal); Supervision (lead); Writing‐review & editing (equal).

## Supporting information

Appendix S1

## Data Availability

Data and relevant code will be stored on the data repository Tropical Data Hub through James Cook University (https://tropicaldatahub.org/).
