# Supplementary material for: Non‐reef habitats in a tropical seascape affect density and biomass of fishes on coral reefs
Source: Ecol Evol. 2020 Nov 19;10(24):13673–86. doi: 10.1002/ece3.6940 (PMC7771147; doi:10.1002/ece3.6940)
Supplement: Supplementary file 1 — Appendix S1 [file ECE3-10-13673-s001.docx]

**Appendix Table 1**. Model output from bootstrapped boosted regression trees for each response variable, showing the mean relative importance for each variable in order of importance, and the 95% lower and upper confidence intervals for relative importance in parentheses. Variables with Dist_ (Distance) or with a value (250, 500, 1000) represent Adjacent Habitat variables calculated at the seascape scale. Variables with (L) are Local Reef scale variables measured on coral reef transects. Bolded values are mean relative importance values greater than 10%, representing high importance variables. MA = macroalgae, SG = seagrass, RF = reef flat, MG = mangrove, Res size = reserve size, Rub = Rubble, SC (L) = soft coral, SC = structural complexity, HC = hard coral, Rob C = robust coral, Frag C = fragile coral, EAM = epilithic algal matrix. For reef fish groups, Acanth = Acanthuridae (Surgeonfishes), Chaet = Chaetodontidae (Butterflyfishes), Serr = Serranidae (Groupers), Pom = Pomacentridae (Damselfishes), Lutj = Lutjanidae (Snapper). *Scarus* and *Chlorurus* are genera from the parrotfish family. Models were run for adults and juveniles (Juv), for density (Den), biomass (Bio), and presence (P/A).

| Model | Species | Stage | Metric | Level | 1 | 2 | 3 | 4 | 5 | 6 | 7 | 8 | 9 | 10 |
| --- | --- | --- | --- | --- | --- | --- | --- | --- | --- | --- | --- | --- | --- | --- |
| 1 | Acanth | Adult | Bio | Crest | **Depth** | **Sand (L)** | **Res size** | **SG1000** | **Dist.Shore** | MA (L) | Dist_MA | Dist_MG | MG500 | Status |
|  |  |  |  |  | **16.4** | **15.7** | **13.9** | **12.0** | **10.6** | 9.1 | 8.9 | 6.6 | 4.5 | 2.3 |
|  |  |  |  |  | (6.6, 32.1) | (3.9, 30.9) | (1.8, 33.5) | (3.5, 23.2) | (2.7, 22.9) | (1.2, 17.9) | (1.9, 20.1) | (1.5, 18.3) | (0.5, 14.6) | (0.1, 5.9) |
| 2 | Acanth | Adult | Bio | Slope | **SC (L)** | **Frag_C (L)** | **MA500** | **SG1000** | **Dist_SG** | **Rub (L)** | Sand500 | Res size | MG500 | Status |
|  |  |  |  |  | **16.9** | **16.1** | **13.3** | **12.7** | **10.7** | **10.6** | 8.9 | 6.6 | 3.8 | 0.4 |
|  |  |  |  |  | (0.8, 38.1) | (3.5, 38) | (1.3, 39.3) | (2.4, 34.7) | (1.5, 23.7) | (0.6, 23.3) | (1.6, 23) | (0.2, 28.9) | (0.3, 12.9) | (0, 1) |
| 3 | Acanth | Adult | Abund | Crest | **Depth** | **Res size** | **Sand (L)** | **Dist_MG** | SG1000 | MA (L) | MG500 | Rub (L) | Dist.Shore | Status |
|  |  |  |  |  | **16.0** | **15.6** | **13.0** | **12.3** | 9.6 | 8.9 | 8.8 | 7.9 | 6.3 | 1.6 |
|  |  |  |  |  | (2.8, 43.3) | (4.4, 34.5) | (2.7, 32.8) | (1.2, 36.3) | (2.1, 24.8) | (3, 18.2) | (1.3, 29) | (1.7, 26.2) | (1.6, 14.5) | (0.4, 4.3) |
| 4 | Acanth | Adult | Abund | Slope | **Frag_C (L)** | **Sand500** | **SG1000** | **Dist_MG** | **Dist_SG** | MA (L) | Res size | Sand (L) | SC (L) | Status |
|  |  |  |  |  | **16.3** | **15.5** | **14.9** | **11.0** | **10.6** | 9.4 | 7.8 | 7.8 | 6.3 | 0.4 |
|  |  |  |  |  | (3.2, 36.4) | (2.6, 34.9) | (1.7, 36.9) | (1.9, 29.1) | (1.4, 25.7) | (0.7, 23.5) | (0.5, 37.4) | (0.4, 27.3) | (1, 19.5) | (0, 2) |
| 5 | Chaet | Adult | Bio | Crest | **MG500** | **Depth** | **MA500** | HC (L) | Sand500 | EAM (L) | Sand (L) | Res size | Dist_SG | Status |
|  |  |  |  |  | **23.6** | **22.1** | **18.9** | 7.6 | 7.1 | 6.9 | 4.7 | 4.7 | 4.3 | 0.2 |
|  |  |  |  |  | (2.1, 55.7) | (3.1, 54.9) | (2.1, 54.4) | (1.3, 18.8) | (1, 24.8) | (1.4, 18.1) | (0.7, 10.4) | (0.2, 14) | (0.5, 16.6) | (0, 0.7) |
| 6 | Chaet | Adult | Bio | Slope | **Depth** | **Dist_MG** | **Rub (L)** | Frag_C (L) | Sand500 | MG500 | CR250 | Dist_SG | Sand (L) | Status |
|  |  |  |  |  | **18.9** | **15.7** | **12.9** | 10.0 | 9.7 | 9.4 | 7.8 | 7.1 | 6.8 | 1.6 |
|  |  |  |  |  | (1.1, 46.7) | (2.2, 48.8) | (1.7, 30.7) | (1.1, 25) | (0.7, 30.5) | (0.6, 36.2) | (1.2, 25.1) | (0.7, 21.2) | (0.9, 19.4) | (0.1, 6.9) |

| 7 | Chaet | Adult | Abund | Crest | **Depth** | **MG500** | **MA500** | Sand (L) | Dist_SG | HC (L) | Sand500 | EAM (L) | Res size | Status |
| --- | --- | --- | --- | --- | --- | --- | --- | --- | --- | --- | --- | --- | --- | --- |
|  |  |  |  |  | **24.6** | **23.3** | **18.1** | 9.4 | 5.8 | 5.8 | 4.9 | 4.3 | 3.5 | 0.2 |
|  |  |  |  |  | (3.4, 62.6) | (5.3, 55.9) | (1.3, 61.2) | (1.2, 24.4) | (0.5, 24.5) | (1.1, 17.2) | (0.9, 20.9) | (0.6, 11.7) | (0.3, 9.5) | (0, 0.8) |
| 8 | Chaet | Adult | Abund | Slope | **MG500** | **Sand500** | **Dist_MG** | **Rub (L)** | **SG1000** | **Depth** | Frag_C (L) | Sand (L) | Dist_SG | Status |
|  |  |  |  |  | **20.1** | **15.8** | **15.7** | **11.4** | **10.7** | **10.3** | 6.2 | 5.0 | 3.7 | 1.3 |
|  |  |  |  |  | (1.8, 56.9) | (1.6, 45.2) | (2.6, 41.7) | (2.3, 36.4) | (1.7, 24.4) | (0.9, 22.7) | (0.7, 16.8) | (1.3, 11.2) | (0.3, 10.1) | (0.1, 3.9) |
| 9 | *Scarus* | Adult | Bio | Crest | **MA (L)** | **MG500** | **Depth** | **Rub (L)** | **HC (L)** | Sand (L) | SC (L) | MA1000 | SC | Status |
|  |  |  |  |  | **17.0** | **14.2** | **13.9** | **13.5** | **10.7** | **10.7** | 9.2 | 4.6 | 3.8 | 2.4 |
|  |  |  |  |  | (5.8, 28.5) | (2.5, 42.7) | (3.9, 32) | (3, 33) | (3.8, 26.1) | (3, 27.9) | (2.5, 18.7) | (0.8, 16.8) | (0.1, 12.7) | (0.2, 8.9) |
| 10 | *Scarus* | Adult | Bio | Slope | **Rob_C (L)** | **Dist_SG** | **MA (L)** | **Sand (L)** | **Rub (L)** | SG250 | MA250 | Dist.Shore | RF500 | Status |
|  |  |  |  |  | **22.1** | **15.6** | **13.7** | **11.3** | **11.0** | 8.0 | 6.6 | 5.8 | 4.4 | 1.6 |
|  |  |  |  |  | (6.3, 43.8) | (3.6, 41.8) | (2.9, 28.6) | (2.8, 22.9) | (2.7, 34.9) | (1.3, 25.3) | (1.2, 18) | (0.9, 17) | (0.6, 15.5) | (0.1, 8.3) |
| 11 | *Scarus* | Adult | Abund | Crest | **MA (L)** | **MG500** | **Depth** | **Sand (L)** | **HC (L)** | MA1000 | Dist_SG | Sand500 | SC | Status |
|  |  |  |  |  | **17.1** | **15.5** | **14.6** | **10.6** | **10.5** | 8.9 | 7.7 | 7.2 | 5.7 | 2.0 |
|  |  |  |  |  | (7.2, 33.3) | (2, 42.8) | (4.7, 30.3) | (2.8, 21.7) | (4.6, 26.5) | (1.1, 30.5) | (1.8, 20.5) | (1.9, 17.1) | (0.2, 19.5) | (0.2, 8.4) |
| 12 | *Scarus* | Adult | Abund | Slope | **EAM (L)** | **Dist_SG** | **MA (L)** | **Frag_C (L)** | **RF250** | SC (L) | Sand (L) | Sand500 | Dist.Shore | Status |
|  |  |  |  |  | **15.0** | **13.7** | **13.6** | **12.6** | **11.1** | 9.0 | 8.3 | 7.9 | 6.9 | 1.9 |
|  |  |  |  |  | (6.5, 30.9) | (3, 33.4) | (3.1, 24.3) | (5.2, 24.8) | (3.6, 21.8) | (2.3, 21.5) | (3.5, 19.8) | (2, 22) | (1.6, 16.2) | (0.1, 5.6) |
| 13 | *Scarus* | Juv | Abund | Crest | **Frag_C (L)** | **Dist_MG** | Depth | SG1000 | Sand (L) | SC (L) | Sand500 | MG500 | Dist_MA | Status |
|  |  |  |  |  | **41.8** | **23.5** | 7.6 | 6.2 | 5.1 | 4.7 | 4.7 | 3.0 | 2.6 | 0.6 |
|  |  |  |  |  | (11.3, 76.9) | (1.6, 56.7) | (1.4, 24.5) | (0.5, 29.1) | (0.8, 21.1) | (0.9, 10.1) | (0.5, 18.8) | (0.2, 12.3) | (0.3, 7.3) | (0, 2.3) |
| 14 | *Scarus* | Juv | Abund | Slope | **Frag_C (L)** | **Sand500** | **MA (L)** | **Sand (L)** | Dist_MG | Depth | EAM (L) | SG1000 | MG500 | Status |
|  |  |  |  |  | **26.4** | **20.4** | **12.4** | **10.2** | 7.9 | 7.8 | 7.1 | 4.7 | 1.6 | 1.5 |
|  |  |  |  |  | (7.8, 58.7) | (5.1, 51.5) | (3.1, 26) | (3.4, 22.8) | (0.6, 27.3) | (0.5, 22.7) | (1.4, 19.1) | (1.2, 16) | (0.1, 5.6) | (0.1, 7.4) |
| 15 | *Chlorurus* | Adult | Bio | Crest | **Frag_C (L)** | **Dist_SG** | **MA (L)** | **MG500** | Res size | Sand500 | SC | SG1000 | Dist_MG | Status |
|  |  |  |  |  | **26.0** | **14.7** | **12.7** | **10.4** | 9.5 | 8.5 | 6.6 | 6.5 | 4.6 | 0.5 |
|  |  |  |  |  | (10.1, 47.7) | (1.5, 52.5) | (2.2, 28.8) | (1.8, 29.9) | (1, 23.9) | (1.7, 19.3) | (0.2, 22.7) | (1.1, 19.1) | (0.6, 11.5) | (0, 1.7) |
| 16 | *Chlorurus* | Adult | Bio | Slope | **Rob_C (L)** | **Sand (L)** | **Rub (L)** | **Sand500** | SG1000 | MA (L) | MG500 | SC | Res size | Status |
|  |  |  |  |  | **24.5** | **19.1** | **11.1** | 9.6 | 9.2 | 8.8 | 6.8 | 5.2 | 5.1 | 0.6 |
|  |  |  |  |  | (9.1, 45.2) | (4.5, 37.4) | (2.6, 20.3) | (2.4, 22.3) | (2.1, 28.6) | (2.2, 21.5) | (1.4, 16) | (0.9, 16.6) | (0.4, 18.5) | (0, 2.8) |
| 17 | *Chlorurus* | Adult | Abund | Crest | **Frag_C (L)** | **SG1000** | **Dist_SG** | **MA (L)** | Depth | MG500 | Res size | SC | Dist_MG | Status |
|  |  |  |  |  | **20.0** | **16.8** | **16.8** | **13.7** | **12.5** | 5.8 | 5.1 | 5.0 | 3.9 | 0.4 |
|  |  |  |  |  | (4.3, 52.5) | (1.9, 49.2) | (1.7, 41.3) | (3, 27.2) | (2.1, 30.2) | (0.4, 20.3) | (0.4, 13.7) | (0.1, 20.6) | (0.7, 15.8) | (0, 1.5) |
| 18 | *Chlorurus* | Adult | Abund | Slope | **Sand (L)** | **Rob_C (L)** | **MA (L)** | **Sand500** | SG500 | Dist_MG | Dist.Shore | SC | RF250 | Status |
|  |  |  |  |  | **17.1** | **17.0** | **15.6** | **14.3** | 9.7 | 8.4 | 5.6 | 5.6 | 4.9 | 1.8 |
|  |  |  |  |  | (5, 33.5) | (5.4, 32.6) | (2.5, 44.1) | (2.1, 39.5) | (1.8, 27.4) | (1.4, 21.9) | (1.9, 13) | (0.6, 14.8) | (1.6, 12.6) | (0.1, 8.7) |
| 19 | *Chlorurus* | Juv | Abund | Crest | **Sand500** | **Sand (L)** | **Frag_C (L)** | **SC (L)** | Res size | SG500 | Dist_MA | MG1000 | Dist_MG | Status |
|  |  |  |  |  | **31.2** | **17.4** | **10.1** | **8.5** | 7.0 | 6.4 | 6.1 | 5.3 | 4.8 | 3.2 |
|  |  |  |  |  | (2.6, 68.8) | (2.9, 48.2) | (1.1, 36.5) | (1.2, 22.3) | (0.4, 24.9) | (0.9, 14.8) | (0.7, 21.6) | (0.3, 23.4) | (0.1, 14.8) | (0.1, 14.1) |
| 20 | *Chlorurus* | Juv | Abund | Slope | **Sand1000** | **Dist_SG** | **Sand (L)** | **Rub (L)** | Depth | MG1000 | Dist.Shore | Dist_MG | Dist_MA | Status |
|  |  |  |  |  | **22.6** | **22.3** | **19.8** | **11.3** | 8.4 | 4.0 | 3.9 | 3.0 | 2.9 | 1.7 |
|  |  |  |  |  | (0.3, 77.7) | (0.8, 74.2) | (0.2, 62) | (0.1, 39.5) | (0.3, 49) | (0.1, 25.8) | (0.1, 18.1) | (0.1, 21.6) | (0.1, 17.9) | (0, 12.5) |
| 21 | Labridae | Adult | Bio | Crest | **MA500** | **Depth** | **EAM (L)** | **HC (L)** | **Sand (L)** | RF250 | Dist_MA | SG1000 | SC | Status |
|  |  |  |  |  | **20.8** | **14.6** | **13.6** | **12.1** | **11.9** | 9.4 | 7.5 | 4.9 | 3.5 | 1.7 |
|  |  |  |  |  | (5.2, 44.6) | (4, 32.6) | (3, 30.9) | (1.7, 34.1) | (2, 40.5) | (1.3, 26.3) | (1.2, 20) | (0.6, 13.6) | (0, 15.7) | (0.1, 6.9) |
| 22 | Labridae | Adult | Bio | Slope | **Sand250** | **Depth** | **EAM (L)** | **MA (L)** | **Rob_C (L)** | **Sand (L)** | RF250 | Dist.Shore | SC (L) | Status |
|  |  |  |  |  | **16.2** | **15.2** | **10.9** | **10.7** | **10.5** | **10.2** | 9.5 | 8.2 | 6.9 | 1.7 |
|  |  |  |  |  | (2.6, 39.1) | (3.8, 33.9) | (2.2, 20.8) | (3.7, 21.6) | (2.8, 25.5) | (2.9, 22.1) | (2.3, 25.5) | (2.3, 19) | (1.4, 18.2) | (0.2, 4.9) |
| 23 | Labridae | Adult | Abund | Crest | **Dist_MG** | **HC (L)** | **Depth** | **EAM (L)** | Sand (L) | SG1000 | Dist_MA | Sand250 | MG500 | Status |
|  |  |  |  |  | **24.4** | **19.9** | **18.7** | **12.4** | 10.0 | 4.9 | 3.6 | 2.9 | 1.8 | 1.4 |
|  |  |  |  |  | (3.2, 56.6) | (3.1, 48.3) | (1.9, 39.1) | (3.3, 29.5) | (1.2, 29.9) | (0.5, 14) | (0.5, 10) | (0.4, 10.1) | (0.1, 9) | (0.1, 4.6) |
| 24 | Labridae | Adult | Abund | Slope | **HC (L)** | **Sand (L)** | **Depth** | EAM (L) | MA (L) | SC (L) | SG1000 | RF250 | Sand500 | Status |
|  |  |  |  |  | **24.8** | **18.6** | **13.8** | 8.9 | 7.7 | 7.4 | 6.5 | 5.8 | 5.3 | 1.3 |
|  |  |  |  |  | (7.6, 47.1) | (6.5, 43.4) | (4.5, 34.6) | (2.3, 16.9) | (1.7, 18) | (1.7, 12.7) | (2, 13.3) | (1.3, 16.1) | (0.7, 14.3) | (0.1, 5.3) |
| 25 | Labridae | Juv | Abund | Crest | **Sand500** | **Frag_C (L)** | **SG1000** | **Sand (L)** | MA (L) | Rub (L) | Dist_SG | SC | MA500 | Status |
|  |  |  |  |  | **28.1** | **18.4** | **13.8** | **12.8** | 6.4 | 6.1 | 4.8 | 4.2 | 2.7 | 2.6 |
|  |  |  |  |  | (6.1, 55) | (2, 48.7) | (1.9, 36.7) | (3.6, 30.7) | (1.4, 17.7) | (1.4, 14.3) | (1, 12.3) | (0.1, 11.3) | (0.7, 6.4) | (0.6, 8.5) |
| 26 | Labridae | Juv | Abund | Slope | **MA1000** | **Sand (L)** | **MA (L)** | Dist_SG | Frag_C (L) | Rub (L) | Dist.Shore | Sand250 | SG500 | Status |
|  |  |  |  |  | **26.6** | **16.1** | **14.0** | 9.8 | 9.4 | 8.0 | 5.7 | 5.2 | 4.4 | 0.7 |
|  |  |  |  |  | (7.5, 45.6) | (4.6, 36) | (2.6, 37.8) | (2, 29.9) | (2.6, 18.1) | (1.9, 19.2) | (0.9, 14.4) | (1.8, 11) | (0.7, 21.6) | (0.1, 2) |
| 27 | Pom | Adult | Abund | Crest | **Depth** | **Frag_C (L)** | **EAM (L)** | **Dist.Shore** | MA (L) | CR250 | MA250 | Sand250 | Dist_MA | Status |
|  |  |  |  |  | **35.4** | **14.2** | **12.0** | **10.2** | 8.6 | 6.0 | 5.9 | 3.8 | 2.9 | 1.0 |
|  |  |  |  |  | (11.4, 61.4) | (4.2, 34.2) | (2.3, 26.2) | (2.4, 22.1) | (1.4, 22.6) | (1.1, 16.6) | (1.5, 15.3) | (0.4, 8.5) | (0.5, 8.2) | (0.1, 4.5) |
| 28 | Pom | Adult | Abund | Slope | **Dist_MG** | **Dist_SG** | **Rub (L)** | **Depth** | **Dist.Shore** | **MA (L)** | CR250 | Res size | MG500 | Status |
|  |  |  |  |  | **18.4** | **17.7** | **14.7** | **13.8** | **13.5** | **11.5** | 4.7 | 3.0 | 2.1 | 0.6 |
|  |  |  |  |  | (2.4, 41.9) | (1.6, 47.6) | (4.7, 32.3) | (3.4, 42.3) | (1.3, 56.1) | (1.8, 28.2) | (0.7, 16.3) | (0.2, 10.3) | (0.2, 10.1) | (0, 2.4) |
| 29 | Lutj | Adult | P/A | Crest | **Sand (L)** | **Frag_C (L)** | **Dist.Shore** | MA1000 | RF250 | Sand250 | SC | SG500 | Res size | Status |
|  |  |  |  |  | **25.0** | **20.9** | **12.0** | 8.1 | 7.8 | 7.4 | 7.2 | 6.7 | 4.7 | 0.3 |
|  |  |  |  |  | (4.8, 64.2) | (2.9, 55) | (1.9, 41.3) | (1.6, 19.9) | (1, 24.2) | (1.2, 22.7) | (0, 29.5) | (0.9, 17) | (1, 12.4) | (0, 1.5) |
| 30 | Lutj | Adult | P/A | Slope | **SC (L)** | **Rub (L)** | **Sand (L)** | Frag_C (L) | CR500 | Depth | MA (L) | Dist.Shore | RF1000 | Status |
|  |  |  |  |  | **21.4** | **20.3** | **19.4** | 8.4 | 7.7 | 7.2 | 5.9 | 3.9 | 3.8 | 1.9 |
|  |  |  |  |  | (4.5, 53.8) | (3.5, 44.2) | (3.5, 38.2) | (1, 31.4) | (1.3, 23.2) | (1.8, 18.5) | (1.4, 15) | (0.5, 14.6) | (0.3, 13) | (0.2, 5.9) |
| 31 | Serr | Adult | P/A | Crest | **Dist_SG** | **MG500** | **HC (L)** | Res size | SC (L) | SG250 | Dist.Shore | RF500 | Dist_MG | Status |
|  |  |  |  |  | **32.4** | **19.7** | **11.6** | 8.3 | 7.2 | 6.4 | 5.5 | 4.9 | 3.2 | 0.7 |
|  |  |  |  |  | (4.5, 64.2) | (3.1, 46.8) | (1.8, 25.7) | (0.6, 30.1) | (1.1, 16.9) | (0.8, 23.2) | (0.7, 14.2) | (0.7, 14.9) | (0.4, 9.2) | (0, 2.8) |
| 32 | Serr | Adult | P/A | Slope | **MA (L)** | **EAM (L)** | **RF500** | **MG500** | Res size | SG250 | Sand250 | SC | Dist_MG | Status |
|  |  |  |  |  | **22.1** | **15.1** | **12.9** | **12.0** | 9.5 | 9.4 | 6.6 | 5.9 | 5.4 | 1.2 |
|  |  |  |  |  | (7.9, 42.9) | (3.5, 35.4) | (1, 38.8) | (2.7, 29.4) | (0.9, 21.8) | (1.7, 25.4) | (1.1, 17.5) | (0.1, 18.3) | (0.9, 18.7) | (0.1, 4.1) |
